# Predictors of full functional recovery in endovascularly treated patients with aneurysmal subarachnoid hemorrhage

**DOI:** 10.3906/sag-2103-3

**Published:** 2021-08-30

**Authors:** Fatih UZUNKAYA, Ayşegül İDİL SOYLU

**Affiliations:** 1 Department of Radiology, Faculty of Medicine, Ondokuz Mayıs University, Samsun Turkey

**Keywords:** Subarachnoid hemorrhage, cerebral aneurysm, outcome, prediction

## Abstract

**Background/aim:**

The knowledge of factors influencing functional outcomes after aneurysmal subarachnoid hemorrhage (ASH) has significantly increased in recent decades, still not enough. We aimed to identify the predictors of full functional recovery (FFR) in endovascularly treated patients with ASH.

**Materials and methods:**

A retrospective review was performed of adult patients who underwent endovascular treatment for ASH in a 5-year period. The association was evaluated of variables with FFR, defined as a modified Rankin Scale score of 0 or 1 at a 3-month follow-up.

**Results:**

This study included 204 patients with a percentage of FFR of 62.7%. On univariate analysis, the following variables were associated with FFR: younger age, male sex, no history of hypertension, posterior circulation aneurysm, better modified-Fisher grade (mFG), better Hunt-Hess grade, better Glasgow Coma score, lower platelet-to-lymphocyte ratio (PLR), lower neutrophil-to-lymphocyte ratio (NLR), and higher platelet-to-neutrophil ratio (PNR). On multivariate analysis, younger age (OR = 0.95, 95% Cl = 0.92–0.98, *p* = 0.003), better mFG (OR = 0.66, 95% Cl = 0.48–0.97, *p* = 0.03), lower PLR (OR = 0.993, 95% Cl = 0.990–0.997, *p* = 0.001), lower NLR (OR = 0.89, 95% Cl = 0.83–0.95, *p* = 0.01) and higher PNR (OR = 1.08, 95% Cl = 1.01–1.10, *p* = 0.01) showed the strongest association with FFR.

**Conclusion:**

With the administration of endovascular treatment, most of the patients with ASH can return to a normal productive life. Younger age, better mFG, lower PLR and NLR, as well as higher PNR, increase the likelihood of FFR.

## 1. Introduction

Thanks to the advances in supportive neurocritical care and refinements either in surgical or endovascular treatment (EVT), the prognosis of patients with a ruptured cerebral aneurysm has remarkably improved over time [14]. To achieve a much better outcome, many studies have been performed to test the influence of various factors on the functional, as well as cognitive outcomes after aneurysm rupture, mainly regarding consequential disability [57]. However, no consensus has emerged among researchers over the validity of factors tested, perhaps because of the differences in research design, as well as the ethnicity of the study population. Besides, new variables have been added to the checklist in recent years, including novel inflammatory markers [8]. Given that the therapeutic goal should be the best outcome in patients with aneurysmal subarachnoid hemorrhage [9], in this study, we aimed to identify the predictors of full functional recovery in a contemporary series of endovascularly treated patients with a ruptured cerebral aneurysm. 

-- 

## 2. Materials and methods 

### 2.1. Study population

After approval of the local Ethical Committee, a retrospective chart review was conducted using the electronic medical record identifying adult patients who underwent an EVT for cerebral aneurysm, at our angiography unit during the period between January 2015 to May 2020. Among them, patients with an unruptured aneurysm, fusiform aneurysm or unsuccessful treatment were excluded from this study (Figure).

**Figure 1:**
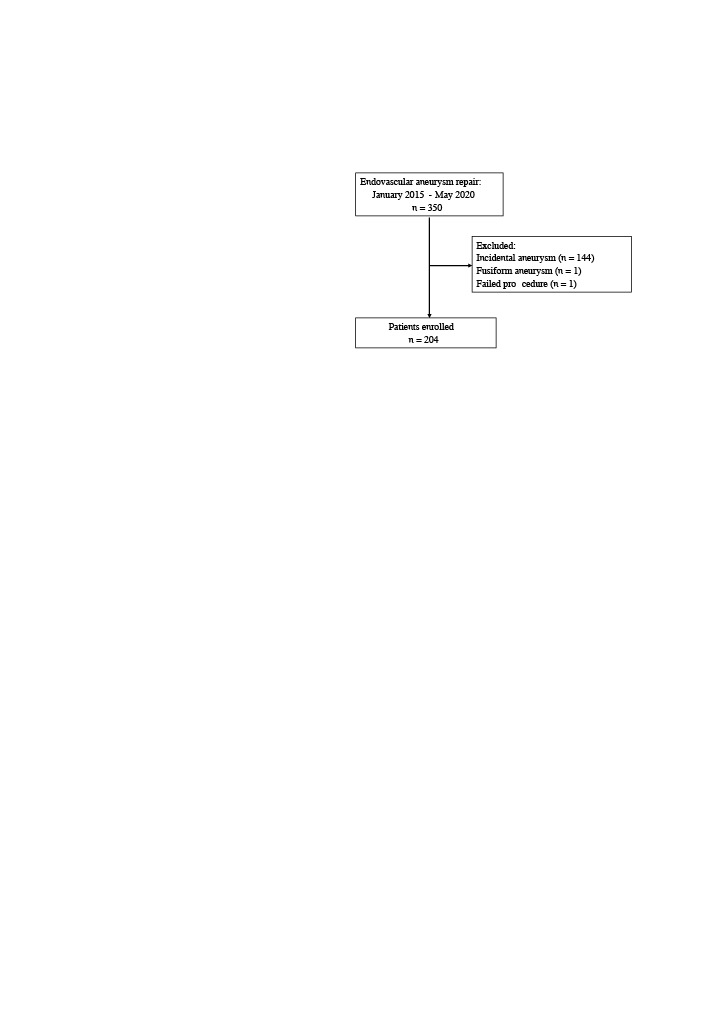
Flow chartshowing the selection ofthe study population.

### 2.2. Study protocol

Demographic characteristics and previous medical history (i.e., history of hypertension, diabetes, coronary artery disease and cerebrovascular disease) of the patients were identified. In addition, the location and size of the aneurysms were inspected. Location of the aneurysms was mainly assessed in two groups: anterior or posterior circulation aneurysms. Aneurysmal size was measured in maximum diameter, either on preoperative CT angiography or intraoperative 3D rotational angiography. The size of fusiform aneurysms was not included in the evaluation. The amount of subarachnoid blood was graded using the modified Fisher , using the first available noncontrast enhanced CT scan. The Hunt & Hess grade and the Glasgow coma scale score of the patients were the other clinical scores identified (Scale-Scale see Table 1 for the definition of all these three scales used in the study) [10]. The period elapsed from aneurysm rupture to EVT was calculated, as well. ndovascular technique used for the embolizations was investigated. Procedural complications, such as aneurysm perforation and thromboembolism, were identified. Thromboembolic events were assessed in two subgroups: angiographically evident thromboembolism and microemboli. The latter included the patients without angiographic evidence of thromboembolism during EVT but with neurological deficit recognized upon recovery of the general anesthesia. Intraoperative administrations of intraarterial tirofiban hydrochloride (HCl) and tissue plasminogen activator (tPA) were identified, too. Novel inflammatory markers, such as platelet-to-lymphocyte ratio (PLR), platelet-to-neutrophil ratio (PNR) and neutrophil-to-lymphocyte ratio (NLR), were the last parameters investigated. All ratios were calculated by proportioning the absolute counts of each type of cell at admission. 

**Table 1:** Definitions of the scales used in this study.

The modified Fisher grade		
Grade	CT manifestations	
1	No hemorrhage	
2	Extensive bleeding but no blood clots, the thickness of blood products <1 mm	
3	The thickness of the blood products >1 mm	
4	Intracerebral hematoma or intraventricular hemorrhage	
The Hunt-Hess grade		
Grade	Early prognosis	
1	No symptoms, or only mild headache and neck stiffness	
2	A heavy, stiff neck, in addition to oculomotor nerve palsy, without any symptoms of the nervous system	
3	Mild disturbance of consciousness, restlessness, and mild symptoms of brain	
4	Semicoma, hemiplegia, decerebrate rigidity, and autonomic nervous disorder at early stage	
5	Deep coma, decerebrate rigidity, endangered state	
The Glasgow coma scale		
Response	Scale	Score
Eye opening response	Eyes open spontaneously	4
	Eyes open to verbal command, speech, or shout	3
	Eyes open to pain (not applied to face)	2
	No eye opening	1
Verbal response	Oriented	5
	Confused conversation, but able to answer questions	4
	Inappropriate responses, words discernible	3
	Incomprehensible sounds or speech	2
	No verbal responses	1
Motorresponse	Obeys commands for movement	6
	Purposeful movement to painful stimulus	5
	Withdraws from pain	4
	Abnormal (spastic) flexion, decorticate posture	3
	Extensor (rigid) response, decerebrate posture	2
	No motor response	1

Please Ethree Based on the information provided in the medical records, the functional outcomes of the patients were evaluated using the modified Rankin (mRS) score. As the primary outcome measure, we defined full functional recovery at three months as an mRS score of 0–1 (0 = no symptoms at all, 1 = no significant disability despite symptoms and able to carry out all usual duties and Scale activities), representing full functional recovery (see Table 2Please for the definition of mRS) [11]. 

**Table 2:** The modified Rankin scale.

Score	Description
0	No symptoms
1	No significant disability. Able to carry out all usual activities, despite some symptoms
2	Slight disability. Able to look after own affairs without assistance, but unable to carry out all previous activities
3	Moderate disability. Requires some help, but able to walk unassisted
4	Moderate severe disability. Unable to attend to own bodily needs without assistance, and unable to walk unassisted
5	Severe disability. Requires constant nursing care and attention, bedridden, incontinent
6	Dead

### 2.3. Statistical analysis 

Statistical Package for Social Sciences for Windows, 21.0 (SPSS, Chicago, IL, USA) was used for statistical analysis. For continuous variables, data summaries were expressed as mean ± standard deviation, with a median value in brackets. For categorical variables, frequencies (percentage) were reported. To analyze whether the variables assessed in the study could cause a difference in the outcome, the MannWhitney U test was used for continuous variables or the chi-square test for categorical variables. Univariate and multivariate logistic regression analyses were used to evaluate the associations between the variables specified in the study protocol and the mRS score of 0 to 1 (best/not) as a binary outcome. A p-value of less than 0.05 was considered statistically significant.

## 3. Results

In this study, 204 patients were assessed. The mean age of the patients was 55.4 ± 14.2 years. Of the 204 patients, 119 (58.3%) were women. In 91 (44.6%) patients, there was at least one chronic comorbid disease. The frequencies of hypertension, diabetes, coronary artery disease and cerebrovascular disease were 53.9%, 7.4%, 9.3% and 1.9%, respectively. The most common location of the aneurysms was anterior communicating artery (43.6%). The mean aneurysmal size was 6.0 ± 3.0 . The means of two subarachnoid hemorrhage grading scales of modified Fisher and Hunt & Hess were 2.1 ± 1.1 and 1.5 ± 0.9, respectively. The mean Glasgow score was 13.8 ± 2.3. The mean interval between the aneurysm rupture and the treatment was 6.1 ± 7.5 days. Primary coiling was the most common technique used for embolization (67.2%), which was followed by balloon-assisted coiling (13.2%), stent-assisted coiling (10.2%), flow diverter implant (7.8%)millimetersComa and others (1.6%), respectively. The frequency of procedural complications was 17.6: aneurysm perforation in six patients (2.9%), angiographically evident thromboembolism in 26 patients (12.7%) and microemboli in four patients (1.9%). It was detected that tirofiban HCl was intraoperatively administered in 30 (14.7%) patients because of the following purposes: mainly to dissolve blood clots or to prevent clotting due to a stent which had to be implanted without preoperative antiplatelet loading. In 12 (5.9%) patients, tissue plasminogen activator was intraoperatively administered alone or in combination with tirofiban HCL to dissolve blood clots. The means of PLR, PNR and NLR were 216.1 ± 13.1, 25.8 ± 11.7 and 9.8 ± 6.4, respectively. Full functional recovery was seen in 128 (62.7%) patients. Results of the parametric and nonparametric tests are summarized in Table 3. percent

**Table 3:** Comparison of variables according to functional recovery at 3 months.

Variable	mRS score of 0–1(n = 128)	mRS score of 2–6(n = 76)	p value
Mean age ± SD (median) (years)	52.1 ± 13.4 (54)	60.9 ± 13.9 (60)	<0.001
Female sex	67 (52.3%)	52 (68.4%)	0.03
Hypertension history	60 (46.8%)	50 (65.7%)	0.01
Anterior circulation aneurysm	111 (86.7%)	73 (96%)	0.05
Mean aneurysmal size ± SD (median) (mm)	6.0 ± 2.9 (5.6)	6.0 ± 3.3 (5)	0.33
Mean mFisher grade ± SD (median)	1.9 ± 1.0 (2)	2.6 ± 1.0 (2.5)	<0.001
Mean Hunt & Hess grade ± SD (median)	1.3 ± 0.7 (1)	1.9 ± 1.1 (1)	0.001
Mean Glasgow Coma score ± SD (median)	14.3 ± 1.6 (15)	13.1 ± 3 (14)	<0.001
Mean interval ± SD (median) (days)	6.4 ± 7.4 (4)	5.7 ± 7.6 (3)	0.22
Coil embolization	87 (67.9%)	50 (65.7%)	0.86
Procedural complication	18 (14.0%)	18 (23.6%)	0.12
Tirofiban HCl therapy†	14 (10.9%)	16 (21.0%)	0.07
tPA therapy	5 (3.9%)	7 (9.2%)	0.21
Mean PLR ± SD (median)	194.8 ± 109.8 (173.2)	251.8 ± 110.3 (240.7)	<0.001
Mean PNR ± SD (median)	28.4 ± 13.0 (25.7)	21.5 ± 7.5 (19.2)	<0.001
Mean NLR ± SD (median)	8.1 ± 5.9 (7.0)	12.6 ± 6.3 (11.5)	<0.001

Results of the univariate analyses are summarized in Table 4. The following variables were associated with better chances of achieving full functional recovery: younger age, male sex, no history of hypertension, posterior circulation aneurysm, better modified Fisher and Hunt & Hess grades, better Glasgow score, lower PLR, lower NLR and higher PNR. According to multivariate analysis, the following five variables were the most strongly associated with full functional recovery: younger age, better modified Fisher grade, lower PLR, lower NLR and higher PNR (Table 5). 

**Table 4:** Univariate analyses demonstrating the association of variables with full functional recovery (mRS score of 0–1).

Variable	OR (95% CI)	p value
Age (years)	0.95 (0.93–0.97)	<0.001
Female sex	0.50 (0.28–0.91)	0.02
Hypertension history	0.45 (0.25–0.82)	0.009
Anterior circulation aneurysm	0.26 (0.07–0.94)	0.04
mFisher grade	0.54 (0.40–0.70)	<0.001
Hunt & Hess grade	0.56 (0.41–0.78)	<0.001
Glasgow coma score	1.26 (1.09–1.46)	0.001
PLR	0.99 (0.993–0.998)	0.001
PNR	1.06 (1.03–1.10)	<0.001
NLR	0.88 (0.83–0.93)	<0.001

**Table 5:** Multivariate analyses demonstrating independent associations with full functional recovery (mRS score of 0–1)*

Variable	OR (95% CI)	p value
Age (years)	0.95 (0.92–0.98)	0.003
mFisher grade	0.66 (0.48–0.97)	0.03
PLR	0.993 (0.990–0.997)	0.001
PNR	1.08 (1.01–1.10)	0.01
NLR	0.89 (0.83–0.95)	0.01

Cl = confidence interval, mFisher = modified Fisher, mRS = modified Rankin scale, NLR = neutrophil-to-lymphocyte ratio, OR = odds ratio, PLR = platelet-to-lymphocyte ratio, PNR = platelet-to-neutrophil ratio.

## 4. Discussion

The prognosis of patients with aneurysmal subarachnoid hemorrhage is a highly studied research topic. However, achieving full functional recovery is an aspect that has been rarely examined. There are only few studies focusing on the prediction of full functional outcomes, which has remained under-researched [9,12]. Instead, the poor outcome has been the target of most studies. This is perhaps because of the old prejudice; aneurysmal subarachnoid hemorrhage is a crippling disease anyway. Hence, a less than moderate disability has not been regarded as a failure. Given that the survival rate has significantly increased, and the prognosis of such patients has improved over time thanks to the advances in supportive neurocritical care and refinements in surgical and endovascular treatment, a paradigm shift is needed, whereby the primary outcome measure after aneurysmal bleeding should be fully functional recovery rather than functional independent status [13]. Having achieved such an outcome in almost two-thirds of the patients in this study, the suggestion has shown to be realistic. 

The modified Rankin , as well as the Glasgow outcome scale, has mainly been used to measure functional outcomes in patients who underwent aneurysm repair at either short-term or long-term follow-up. To our knowledge, this is the first study focusing on full functional outcomes at short-term follow-up. Therefore, it is hard to compare our results directly with other researches. In our study, the frequency of the patients who recovered full function was almost the same (62.7% vs. 63.3%) compared with findings from Pegoli et al.’s study [9]; however, in which the follow-up duration was one year. Unlike them, we found that younger age, male , no history of hypertension and posterior circulation aneurysm were associated with full functional recovery. In their study, coil embolization was associated with full functional (excellent, in their words) outcome. However, we only saw a trend towards a higher rate of such an outcome with coil embolization. In another study published by Giede-Jebbe et al. [14], the findings showed that lower age, as well as no history of hypertension, was associated with a favorable outcome. Although ruptured posterior circulation aneurysms were shown to have a poor prognosis and a higher rate of death or severe disability due to the deeper position and more complex morphology compared with anterior circulation aneurysms [10], we found an opposite result, which was surprising to us. This may be due to the dominance of the patients with anterior circulation aneurysm in the study group. Aneurysmal size was not a factor related to the outcome in our study, more likely because the sizes of aneurysms in both outcome groups were closer to each other. 

Like in the previous studies, the impact of admission status on the prognosis has been confirmed [1517]. A better modified Fisher grade, as well as a better Hunt & Hess grade, was associated with full functional recovery on the univariate analysis. However, no association of the same strength between Hunt & Hess grade and full functional recovery was detected on the multivariate analysis. According to us, this is because the Hunt & Hess Scale is relatively less objective than the modified Fisher . The differences between grade 1 and 2, as well as between grade 2 and 3, are particularly equivocal on this scale. For instance, the severity of headache and neck stiffness makes the difference between grade 1 and 2 [18]. Since objective grading with this scale can be difficult from time to time, some patients who should be given a higher grade may be given a lower grade [19]. If this was the case in our study population, it might have overshadowed the strength of the association of Hunt & Hess grade with the outcome of the multivariate analysis. Besides, with the advancement in treatment technology as aforementioned above, some of the patients with a Hunt & Hess grade of over 3 have been shown to be able to have a good prognosis, as happened in our cases [10,20]. The latter may have also influenced the result to come out like this. 

The rate of overall procedural complications, including the aneurysm perforations and thromboembolic events, was 17.6% in our study, which was consistent with the results of previous studies [2124]. For instance, Dinç et al. [23]Scalegender-Scale-reported a complication rate of 15.6% in their series, including 481 patients presenting with a ruptured or an unruptured cerebral aneurysm. The complication rate was lower in our cases with full functional recovery compared with the patients with poor prognosis (14% vs. 23.6%). Moreover, in this study, it was detected that the intraoperative administration of tirofiban HCl was more frequent in patients with a 3-month mRS score of 2 or greater. A similar result was seen regarding tPA administration, despite insignificant statistical differences in both circumstances. Contrary to expectations, these results revealed that the occurrence of procedural complications is not an obstacle to a full functional recovery. However, it should not be overlooked that the main underlying factor behind this success is the proper management of the complications. 

Cerebrovascular event-associated immunosuppression is increasingly recognized as a factor worsening functional outcomes after a stroke or intracerebral hemorrhage [8,14,2527]. For instance, Tao et al. [25] reported that PLR and NLR were associated with an unfavorable outcome after subarachnoid hemorrhage. Giede-Jeppe et al. [14] showed a similar relationship regarding higher NLR. Our findings were consistent with the results of these previous studies. We found that lower PLR and NLR, as well as higher PNR, were strongly associated with full functional recovery. Excessive neuroinflammation triggered by hemorrhage-related endothelial injury explains the elevation in white blood cell counts, including neutrophil count [28].-However, the question of why lymphocyte and neutrophil counts are oppositely affected by subarachnoid hemorrhage remains unclear. We could not obtain a satisfactory answer to this question from the literature [14]. In our opinion, this is an interesting phenomenon to study.

This study has several limitations. Some of the variables previously investigated in the literature were not available in the medical records of our cases, such as the history of smoking. Thus, such an analysis could not be performed including them. In addition, we could not evaluate the effects of hemorrhagic vasospasm on the outcome because, at our hospital, surveillance for vasospasm has mainly been conducted with monitoring of clinical signs but without using imaging techniques unless a sudden clinical deterioration emerges. We could not also assess the influence of secondary hydrocephalus on the outcome because we did not have firm standardized criteria for evaluation. Finally, it would be better to report long-term outcomes in such a study. However, since the long-term outcomes of some of the patients included in the study were not yet available, we only reported short-term outcomes by this study. We plan to publish the long-term outcomes of a larger series of patients in the upcoming period.

Returning to normal productive life is possible in most of the endovascularly treated patients with a ruptured cerebral aneurysm. Particularly, patients at a younger age or patients with less severe bleeding are more likely to recover full function. PLR and PNR, as well as NLR, can be used as independent predictors of full functional recovery after aneurysm rupture. 
